# Auranofin Mediates Mitochondrial Dysregulation and Inflammatory Cell Death in Human Retinal Pigment Epithelial Cells: Implications of Retinal Neurodegenerative Diseases

**DOI:** 10.3389/fnins.2019.01065

**Published:** 2019-10-10

**Authors:** Thangal Yumnamcha, Takhellembam Swornalata Devi, Lalit Pukhrambam Singh

**Affiliations:** Department of Ophthalmology, Visual and Anatomical Sciences (OVAS), Wayne State University School of Medicine, Detroit, MI, United States

**Keywords:** neurodegeneration, mitophagy, auranofin, Trx-TrxR, pyroptosis, inflammation, RPE

## Abstract

**Purpose:**

Photoreceptor degeneration occurs in various retinal diseases including age-related macular degeneration (AMD), Retinitis pigmentosa (RP), and diabetic retinopathy (DR). However, molecular mechanisms are not fully understood yet. The retinal pigment epithelium (RPE) forms the outer blood retinal barrier (oBRB) and supplies glucose, oxygen and nutrients from the fenestrated choriocapillaris to photoreceptors for visual function. Therefore, RPE dysfunction leads to photoreceptor injury/death and progression of blinding eye diseases. This study aims to understand the role of the thioredoxin (Trx) and its reductase (TrxR) redox signaling in human RPE dysfunction and cell death mechanism(s) in an *in vitro* system.

**Methods:**

A human RPE cell line (APRE-19) was cultured in DMEM/F12 medium and treated with auranofin (AF – 4 μM, an inhibitor of TrxR) for 4 and 24 h. Mitochondrial and lysosomal function, cellular oxidative stress and NLRP3 inflammasome activity were measured using cell assays, Western blotting, and confocal microscopy. Antioxidants and anti-inflammatory compounds were tested for blocking AF effects on RPE damage. Cell death mechanisms (LDH release to culture media) were determined using necroptosis, ferroptosis and pyroptosis inhibitors. *P* < 0.05 was considered significant in statistical analysis.

**Results:**

Auranofin causes mitochondrial dysfunction (Δψm↓ and ATP↓), oxidative stress (H_2_O_2_↑) and mitophagic flux to lysosomes. Furthermore, the lysosomal enzyme (cathepsin L) activity is reduced while that of pro-inflammatory caspase-1 (NLRP3 inflammasome) is enhanced in ARPE-19. These effects of AF on ARPE-19 are inhibited by antioxidant N-acetylcysteine (5 mM, NAC) and significantly by a combination of SS31 (mitochondrial antioxidant) and anti-inflammatory drugs (amlexanox and tranilast). AF also causes cell death as measured by cytosolic LDH release/leakage, which is not inhibited by either ferrostatin-1 or necrostatin-1 (ferroptosis and necroptosis inhibitors, respectively). Conversely, AF-induced LDH release is significantly reduced by MCC950 and Ac-YVAD-cmk (NLRP3 and Caspase-1 inhibitors, respectively), suggesting a pro-inflammatory cell death by pyroptosis.

**Conclusion:**

The Trx/TrxR redox system is critical for RPE function and viability. We previously showed that thioredoxin-interacting protein (TXNIP) is strongly induced in DR inhibiting the Trx/TrxR system and RPE dysfunction. Therefore, our results suggest that the TXNIP-Trx-TrxR redox pathway may participate in RPE dysfunction in DR and other retinal neurodegenerative diseases.

## Introduction

Retina is a window to the brain (Chiquita et al., 2019). Being a part of the central nervous system, the retina consumes large amounts of glucose and oxygen for its bioenergetics (ATP production), light perception, and visual processing (Country, 2017). The retina also has tight blood-retinal barriers (BRB) and protects the neuroretina from the circulating immune cells and plasma components (Cunha-Vaz et al., 2011). The inner BRB consists of tight junctions of endothelial cells in the blood vessel while the outer BRB consists of a single layer of retinal pigmented epithelium (RPE) and its tight junction proteins. RPE separates the fenestrated choriocapillaris from the neuroretina and functions to transport glucose, oxygen, and nutrients to the retinal outer segments consisting of rod and cone photoreceptors (Campbell and Humphries, 2012; Ivanova et al., 2019). Breakdown of the outer BRB and RPE dysfunction is associated with age-related macular degeneration (AMD) (Handa et al., 2019; Jun et al., 2019) while gene mutation in RPE and photoreceptor cause photoreceptor degeneration and blindness including retinitis pigmentosa (RP) (Dias et al., 2018; Shu and Dunaief, 2018). In these various retinal diseases, RPE dysfunction, photoreceptor injury/death and retinal neurodegeneration leads to blindness. In addition to nutrient exchange, RPE also involves in recycling of the visual pigment (retinoid) to photoreceptors, daily phagocytosis of the photoreceptor outer segment, and synthesis of melanosome (melanin) to absorb excess light in the retina (Biesemeier et al., 2015; Spencer et al., 2019).

On the other hand, breakdown of the inner BRB and formation of new fragile/leaky blood vessels are associated in proliferative diabetic retinopathy (PDR) leading to blindness as well as visual distortion in diabetic macular edema (DME) (Graham et al., 2018; Bapputty et al., 2019). Recent studies have further demonstrated that photoreceptor oxidative stress and dysfunction may occur early in diabetics prior to vessel histopathology (Fu et al., 2018; Liu et al., 2019). However, the study of the role of RPE in photoreceptor dysfunction in DR is still limited (Xia and Rizzolo, 2017; Tarchick et al., 2019). Diabetic retinopathy (DR) is the most devastating disease of diabetes mellitus affecting millions of people among the working adult life in the US and around the globe (Cheloni et al., 2019). As the number of people living with diabetes particularly of obesity and type 2 diabetes increases, the incident of DR will escalate several folds in coming decades (Caspard et al., 2018). DR is generally defined by microvascular complications of capillary endothelium and pericytes leading to microaneurysm, iBRB leakage, and fragile new blood vessel formation (neovascularization). Most patients with Type 1 or Type 2 diabetes will develop some forms of DR, beginning with non-proliferative DR (NPDR) then progress to a severe form of proliferative DR (PDR) causing blindness (Xia and Rizzolo, 2017; Fu et al., 2018). Yet, the molecular basis of the pathogenesis is not fully understood and, therefore, no known cure or effective treatment options are available still today.

The retina consumes large amounts of glucose and oxygen to generate energy (ATP) for its visual function via the mitochondrial electron transport chain (ETC) in inner membranes (Country, 2017). During this process, electrons leak out from the ETC, which are captured by molecular oxygen generating reactive oxygen radicals/species (ROS), which damage mitochondrial membrane lipid, proteins, and mtDNA. Damaged mitochondrial are inefficient in ATP but produces more ROS. Mitochondrial ROS and oxidized mtDNA, when released into the cytosol, are recognized as damaged-associated molecular patterns (DAMPs) by cytosolic pattern recognition receptors (PRRs) including toll-like receptors TLR4, TLR9, and the NLRP3 inflammasome. These inflammatory receptors produce and activate inflammatory pro-IL-1β and pro-caspase-1. An assembled NLRP3 inflammasome forms the platform for pro-caspase-1 processing and activation, which in turn is responsible for processing pro-inflammatory IL-1 β and active IL-1 β. Caspase-1 also induces pro-inflammatory cell death by pyroptosis, which involves release of the N-terminal part of gasdermin D (Dubois et al., 2019). The cleaved N-terminus portion of gasdermin D is then inserted into the plasma membrane forming pores resulting in plasma membrane permeabilization. Therefore, removal of the damaged mitochondrial by mitophagy, an autophagic process of degrading damaged mitochondria by lysosomes, is critical for maintaining mitochondria health, bioenergetics, and cell survival (Devi et al., 2019). In addition, the RPE phagocytes the damaged photoreceptor outer segment daily and recycle visual pigment retinol for photoreceptor function. Therefore, the mitochondrial-lysosomal axis in RPE and maintaining its function may play a critical role in photoreceptor function in DR. Hence, enhancing the antioxidant capacity while reducing inflammation at initial stages of the disease may constitute potential therapies (Lin and Beal, 2006; Di Carlo et al., 2012; Stephenson et al., 2018; Bapputty et al., 2019).

One of the genes strongly induced by diabetes and aging in retinal cells and neurons is the thioredoxin-interacting/inhibiting protein (TXNIP) (Perrone et al., 2010; Devi et al., 2012, 2013, 2019; Singh and Perrone, 2013). TXNIP’s actions include binding to and inhibition of the anti-oxidant and thiol reducing capacity of thioredoxin (Trx), thereby, causing cellular oxidative stress, inflammation, and premature cell death (Stephenson et al., 2018). Trx1 and its reductase TrxR1 are present in the cytosol and nucleus while Trx2/TrxR2 redox system is in the mitochondrion. Therefore, Trx/TrxR redox system plays an important role in scavenging reactive oxygen species (ROS) and maintaining reduced state of proteins in their functionally active sites and cell survival (Devi et al., 2017; Stephenson et al., 2018; Booty et al., 2019). Thus, TXNIP inhibition of the Trx/TrxR system causes oxidative stress, mitochondrial dysfunction, and retinal cell death in diabetes (Perrone et al., 2010; Singh and Perrone, 2013). Nonetheless, TXNIP is a multifunctional protein and is also considered as a scaffold protein of the α-arrestin type (Stephenson et al., 2018). Therefore, TXNIP is also involved in interactions with other proteins including glucose transporter 1 and 4, REDD1 (regulated in development and DNA damage responses 1), VEGF-R (vascular endothelial growth factor receptor), and other nuclear proteins that are involved in cell cycle regulation (Devi et al., 2012; Stephenson et al., 2018). Therefore, to further understand a direct effect of the Trx/TrxR redox system in RPE and retinal neurodegenerative diseases, we used in this study, auranofin, a gold compound, which specifically inhibits redox proteins TrxR1 and TrxR2 (Yoshihara et al., 2014). We demonstrate that auranofin causes mitochondrial dysfunction, mitophagic flux and inflammatory pyroptotic cell death in RPE cells in culture. These findings may be relevant to various retinal neurodegenerative diseases where RPE dysfunction plays a causative role in disease progression.

## Materials and Methods

### Tissue Culture Media

DMEM medium was purchased from (Mediatech Inc., Cat #10-014-CM, Manassas, VA, United States) while Ham’s F12 was from Hyclone (Cat # SH30025.01, Logan, UT, United States). Antibiotics and trypsin were also purchased from Hyclone, whereas fetal bovine serum was obstained (Corning, Cat # MT35010CV). Details of other chemicals used in this study are provided in Supplementary Table S1.

### Cell Culture

A human retinal pigment epithelial cell line (ARPE-19), purchased from ATCC (Cat# 2302), was maintained in DMEM/F12 medium (1:1 ratio) containing LG (low glucose, 5.5 mM), 5% fetal bovine serum (FBS), 2% penicillin, and 1% antimycotic in a humidified incubator with 5% CO_2_ at 37°C as described previously (Devi et al., 2013). After reaching ∼80% confluence, the medium was changed to 1% serum overnight. Initially, a concentration and time dependent effect of auranofin (AF) on ARPE-19 function was tested; and subsequently we selected 4 μM and 4–24 h as optimal concentration and time period for the current study. Then, ARPE-19 cells were maintained with or without AF (4 μM) for 4 and 24 h, respectively. Because we observed cell death at 24 h but not at 4 h of AF treatment, we decided to investigate early molecular defects that might lead to demise and determine potential rescue mechanisms. Before treatment with 4 μM AF, we pre-incubated ARPE-19 cells with different concentration of drugs targeting mitochondria and NLRP3 inflammasome for 2 h and they were present throughout the period of incubation.

### Measurement of Mitochondrial Membrane Potential

The mitochondrial membrane potential in ARPE-19 cells was measured using MitoProbeTM JC-1 Assay Kit (Cat# M34152, Life Technologies, Eugene, OR, United States) (Devi et al., 2012, 2013). JC1 dye penetrates the cell and exhibits potential-dependent accumulation in mitochondria. After treatment, ARPE 19 cells were washed with 1× phosphate-buffered saline (PBS). Then, the cells were incubated with 200 μl of 2 μM JC1 dye for 10 min in a 48-well cell culture plate. After incubation, the cells were washed thrice with 1× PBS. The relative fluorescence was measured at Ex529/Em590 nm using (SpectraMax Gemini EM Microplate Reader, Molecular Devices) as per manufacturer’s instructions.

### Measurement of Intracellular ATP

Cellular ATP concentration in ARPE-19 cells was measured using a ATP determination kit from Life Technologies (Cat # A22066, Life Technologies, Eugene, OR, United States) (Devi et al., 2012, 2013). After treatment, ARPE-19 cells were washed twice with 1× PBS. Then, 250 μl of 1× TE Buffer (Tris EDTA, 1× solution, pH 8.0, Cat #BP24731, Thermo Fisher Scientific) was added to each well of a 48-well cell culture plate containing the ARPE-19 cells. The cells were scrapped out and transferred to a 1.5 ml Eppendorf tube. The samples were boiled for 5 min in a water bath. After keeping on ice for 3 min, the samples were centrifuged at 12000 rpm for 10 min at 4°C. Ninety μl of the ATP reaction solution and 10 μl of the sample were mixed and kept protected from light. Relative fluorescence units (RFUs) were measured using luminometer plate reader (SpectraMax L Microplate Reader, Molecular Devices).

### Measurement of Intracellular Reactive Oxygen Species (ROS)

Cellular ROS production in ARPE-19 cells was measured using the CM-H2DCFDA probe (Cat # 6827, Life Technologies, Eugene, OR, United States) as per company’s instructions. After different treatments, ARPE-19 cells were washed twice with 1× PBS and incubated with 200 μl of 10 μM CM-H2DCFDA dye for 30 min at 37°C on 24-well cell culture plate. Following incubation, ARPE-19 cells were washed once in 1× PBS. The fluorescence intensity of CM-H2DXFDA was measured at Ex495/Em517 nm in 1× PBS buffer using a Fluorescence Plate Reader (SpectraMax Gemini EM Microplate Reader, Molecular Devices).

### Assessment of Cathepsin-L Activity

Cathepsin-L activity in ARPE-19 cells was measured using a Magic Red Cathepsin-L detection Kit (Cat #941, Immunochemistry Technologies, Bloomington, MN, United States) as described recently (Devi et al., 2013). Briefly, ARPE-19 cells were incubated with 20 μl Magic Red Cathepsin-L reagent and 480 μl of cell culture media for 1 h. The cells were then washed twice with 1× PBS. The fluorescence intensity of dye was measured at Ex592/Em628 nm using a Fluorescence Plate Reader (SpectraMax Gemini EM Microplate Reader, Molecular Devices).

### Assessment of Caspase-1 Activity

Caspase-1 activity in ARPE-19 cells was measured using a FAM-FLICA Caspase-1 detection kit (Cat #97, Immunochemistry Technologies, Bloomington, MN, United States) in a 24-well cell culture plate as described before (Devi et al., 2013). After treatment with various reagents for a stipulated time period, ARPE-19 cells were washed with wash buffer. Then, the cells were incubated with 10 μl of FLICA solution and 300 μl of freshly replaced 1% serum media for 1 h. Following incubation, the cells were washed twice with 1× PBS. The fluorescence intensity was measured at Ex492/Em520 nm using a Fluorescence Plate Reader (SpectraMax Gemini EM Microplate Reader, Molecular Devices).

### Measurement of Lactate Dehydrogenase Activity (LDH)

Lactate dehydrogenase activity in ARPE-19 cells was measured using Pierce TM LDH cytotoxicity Assay Kit (Cat# 88954, Thermo Fisher scientific). After treatment, 50 μl of the Sample’s cell culture media were mixed with 50 μl of reaction mixture in a 96-well plate and incubated at room temperature for 30 min. The absorbance was measured at 490 nm and 680 nm using SpectraMax plus 384 Microplate Reader (Molecular Devices), as per company’s instructions. The amount of LDH release in the media was calculated by difference in absorbance of 490–680 nm, according to manufacturer’s instructions.

### Western Blotting and Mitophagic Flux

These procedures, both Western blotting and mitophagy analysis, were performed similar to those described (Devi et al., 2017, 2019). Cytosol, mitochondria and nuclear fractions were fractionated using a kit as described before (Devi et al., 2012, 2013). Briefly, for Western blots, 20–30 μg protein extracts were loaded on SDS-PAGE, and proteins were separated, trans-blotted and incubated with appropriate primary antibodies. After overnight incubation with primary antibodies at 40_c_ with shaking, HRP conjugate secondary antibodies were added for 2 h. ECL detected the reactive bands in a FluorChem E Western blot imaging instrument (ProteinSimple, San Jose, California). ImageJ (NIH) was used to quantitate the blots. The source of primary and secondary antibodies and their dilution used are shown in Supplementary Tables S2A,B, respectively.

For mitophagy, Ad-CMV-mt-Keima was expressed in the mitochondrial matrix by transduction for 3 days; then treated with AF with or without inhibitors. Mt-Keima emits a green light (neutral or alkaline pH > 7.0) in mitochondria while, in lysosomes after mitophagic flux, it emits red light at acidic pH (<5.0). Therefore, the same mt-Keima can detect mitophagic flux by a change in color from green to red in living cells. A Zeiss LMS 780 Confocal Microscope captured multiple images from triplicate samples. Similarly, a CMV-LAMP1-mCherry carrying adenovirus was transduced in ARPE-19 cells to examine lysosomal morphology and distribution after the AF treatment.

### Statistical Analysis

Results are represented by means ± SEM of the indicated number of experiments. One-way ANOVA and Bonferroni *post hoc* test determined differences among means in multiple sets of experiments. On the other hand, a comparison between two sets of experiments was analyzed by unpaired two-tailed *t*-test. A *p*-value of <0.05 was considered to be statistically significant.

## Results

### Auranofin Causes Mitochondria-Lysosome Dysfunction and Inflammatory Responses in ARPE-19 Cells

**FIGURE 1:**
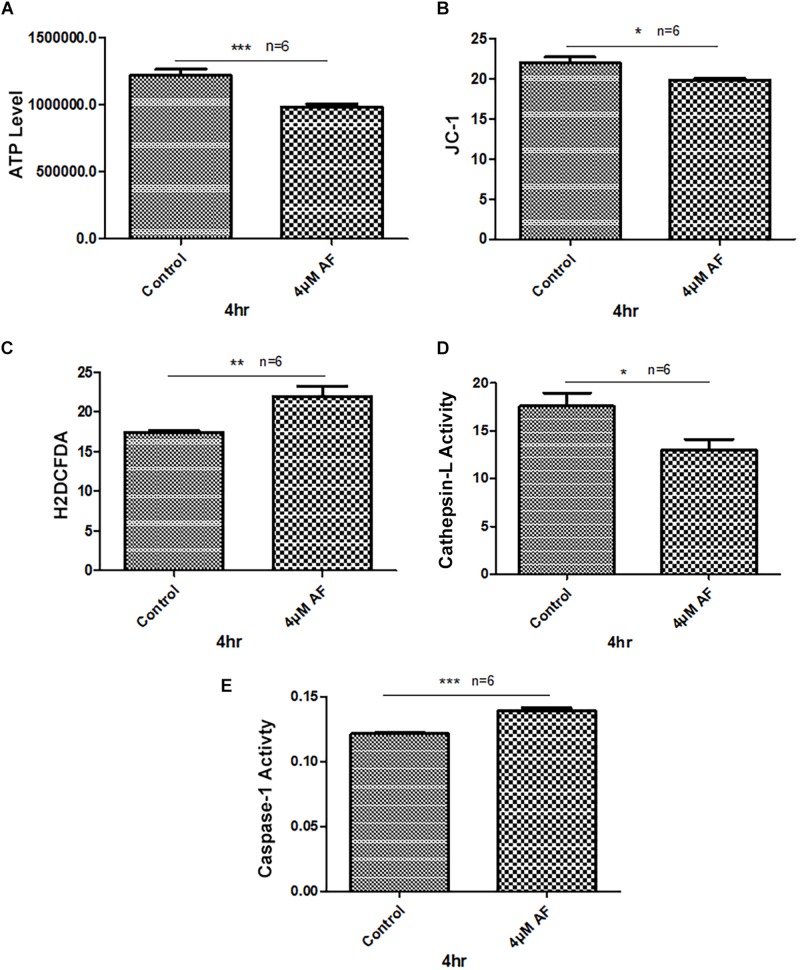
Auranofin causes mitochondrial dysfunction and lysosomal enzyme inactivation in ARPE-19 cells. **(A)** AF treatment (4 μM, 4 h) of ARPE-19 cells reduces cellular ATP level and **(B)** causes mitochondrial membrane depolarization as measured by a reduction in JC1. **(C)** In addition, AF increases cellular ROS levels and **(D)** reduces lysosomal cathepsin L activity. These changes in mitochondrial and lysosomal function are associated with an increase in the activity of proinflammatory enzyme, caspase-1 **(E)**. Significant changes are indicated by *p* values of ^∗^<0.05; ^∗∗^<0.001; and ^∗∗∗^<0.0001; *n* = 6.

**FIGURE 2:**
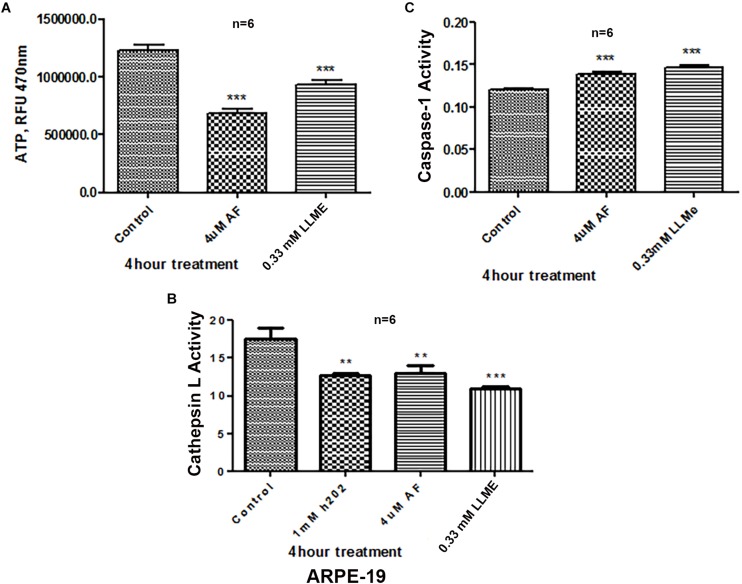
Lysosomal damage reduces ATP levels and activates Caspase-1 activity in ARPE-19 cells. **(A,B)** Treatment with auranofin (AF, 4 μM, 4 h) or lysosomal membrane iononophore (LLMe, 0.33 mM, 4 h) significantly reduces ATP levels and cathepsin L activity. In addition, H_2_O_2_ also reduces cathepsin L activity significantly suggesting a role for oxidative stress. **(C)** Conversely, both AF and LLMe increase pro-inflammatory caspase1 activity in ARPE-19 cells. Significant changes in figures are indicated by *p* values of symbols ^∗∗^<0.001 and ^∗∗∗^<0.0001; *n* = 6 for each experiment.

**FIGURE 3:**
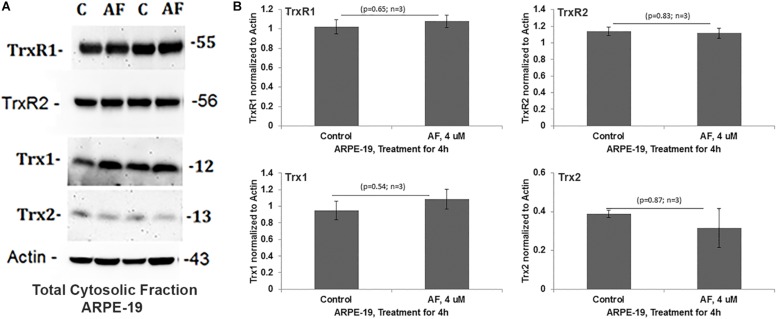
Auranofin does not change the level of redox proteins significantly in ARPE-19 cells. **(A,B)** On Western blots, auranofin treatment does not cause a significant change in protein levels of TrxR1, TrxR2, Trx1, or Trx2 when normalized to actin (*p* > 0.05; *n* = 3).

Treatment of APRE-19 with AF (4 μM) for 4 h reduces significantly the mitochondrial ATP level as well as mediates mitochondrial membrane depolarization as measured by a reduction in JC1 (Figures 1A,B, respectively). In addition, there is an increase in the cellular ROS level as determined by H_2_DCFDA probe (Figure 1C). These results suggest that AF induces mitochondrial dysfunction and cellular oxidative stress in ARPE-19. To further investigate if lysosomal function is also altered due to mitochondrial defects as the mitochondria-lysosome function are closely related, we measured the activity of lysosomal acid hydrolase, cathepsin L. Indeed, we observed that cathepsin L activity is significantly reduced by AF (Figure 1D) and also increases the activity of pro-inflammatory caspase-1 (Figure 1E) suggesting inflammasome activation by the AF treatment. Furthermore, we show that lysosome destabilization by lysosomotropic agent LLME (LeuLeuOMe) also causes a reduction in ATP level along with activation of caspase 1 and inhibition of the cathepsin L activity, which are similar to that observed with AF further supporting that the mitochondria and lysosomal injury in AF action on ARPE-19 cells (Figure 2). Nonetheless, AF does not alter levels of TrxR1 and TrxR2 significantly and similarly no significant change is observed with Trx1 and Trx2, although Trx2 is marginally reduced (Figure 3).

### Auranofin Does Not Evoke mtUPR but Mediates Mitophagic Flux in ARPE-19 Cells

The mitochondrion responses to oxidative stress (i) by increasing the expression of nuclear-encoded mitochondria-targeted chaperones and proteases to counter its oxidative protein stress and misfolding known as the mitochondrial unfolded protein response (mtUPR) (Harper, 2019). (ii) Another mitochondrial stress response is segregation of the damaged part of the mitochondrion by fission involving Drp1 (dynamin related protein 1), then engulfment within a double-membrane autophagosome, which is further targeted to lysosomes for degradation, a process known as mitophagy – autophagy of damaged mitochondria (Pareek and Pallanck, 2018). Nonetheless, we did not observe significant changes in the expression of mitochondrial proteases (LonP and YMEIL1) and chaperones (Tid1/mtHSP40 and PDIA, protein disulfide isomerase A) by AF. Conversely, during the same period of AF treatment, autophagic/mitophagic markers, such as microtubule light-chain LC3BII and adaptors optineurin and p62/Sequestosome1, are reduced within minutes to hours (Supplementary Figure S1), suggesting a mitophagy induction.

**FIGURE 4:**
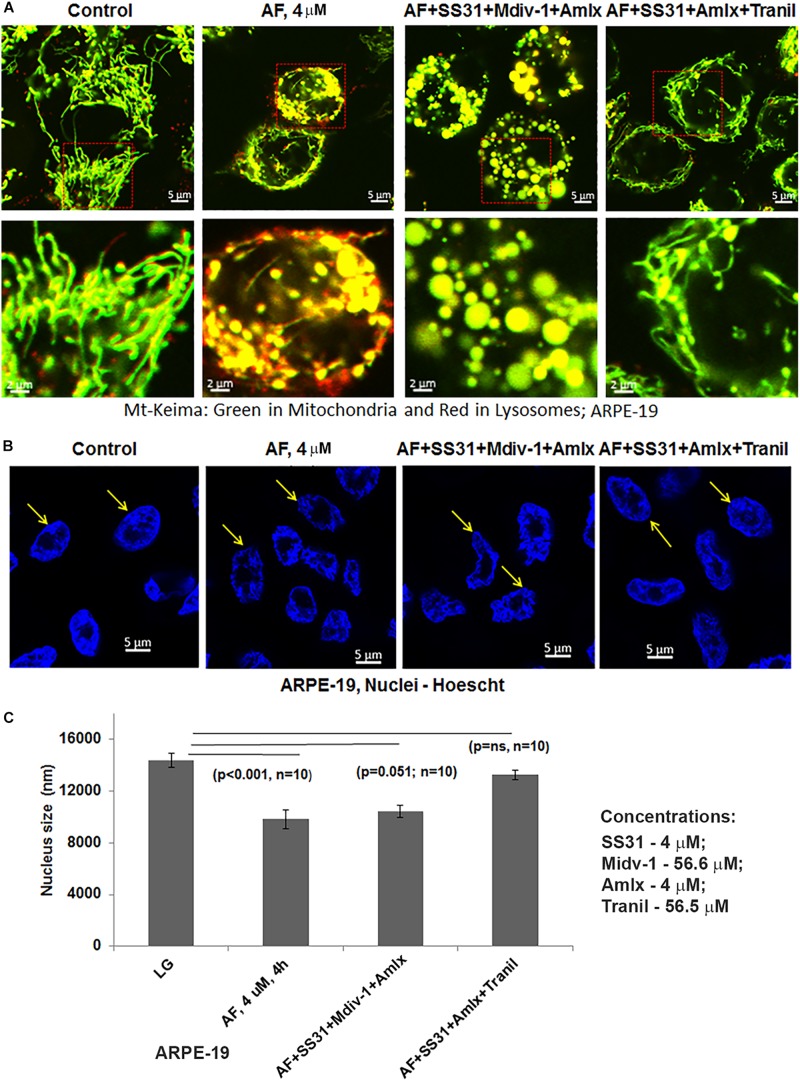
Auranofin causes mitophagic flux and nuclear deformation in ARPE-19 cells. **(A)** AF treatment (4 μM, 4 h) increases the level of red mt-Keima in ARPE-19 cells when compared to the control where green mt-Keima is predominantly observed. Combination treatment of ARPE-19 cells with SS31 + Midiv-1 + Amlexanox (added 2 h before AF) reduces red mt-Keima; however, the treatment also results in the formation of vesiculated (round and enlarged) mitochondria. Conversely, pretreatment with SS31 + Amlexanox + Tranilast prevents AF-induced mitophagy (reduced red mt-Keima); also seen as filamentous green network. A representative of *n* = 3 is shown here at mag. 630×. **(B,C)** AF causes nuclear deformation or chromatin shrinkage as shown by DAPI staining and reduced nuclear diameter compared to the control ARPE-19 (significant decrease, *p* < 0.001 AF vs. Control; *n* = 10 nuclei). Such nuclear changes are prevented by pre-treatment with a combination of SS31 + Amlexanox + Tranilast (p = ns vs. LG, control) but not with SS31 + Midiv-1 + Amlexanox (*p* = 0.051; *n* = 10 vs. LG, control).

**FIGURE 5:**
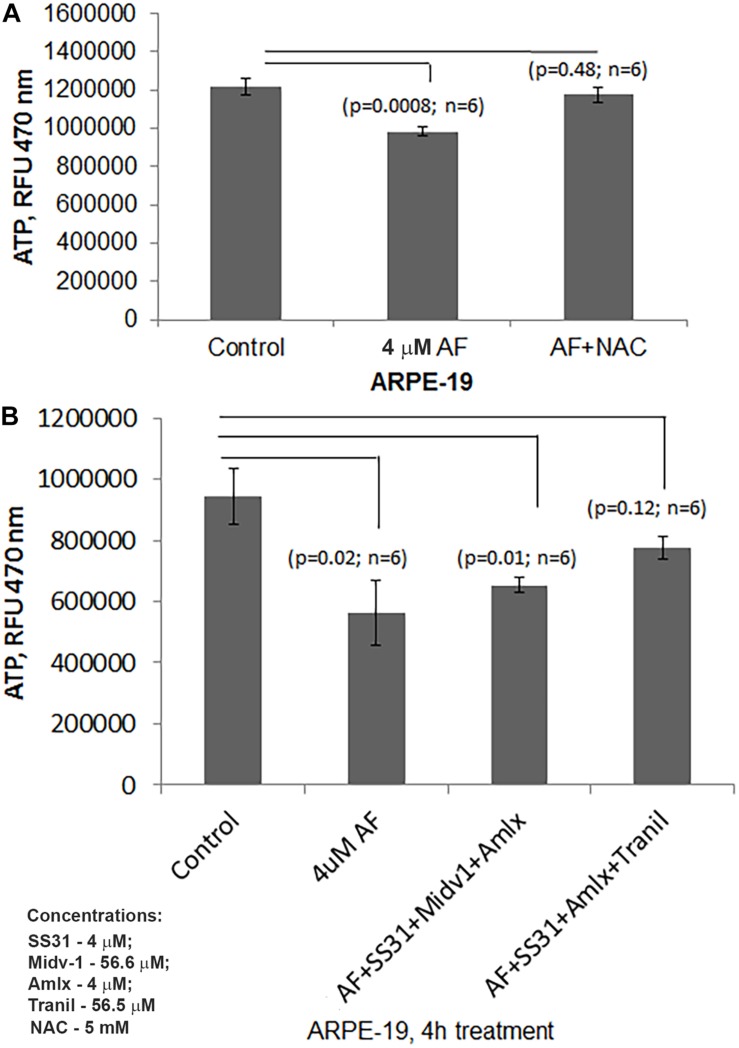
AF reduced ATP levels are restored by combination treatment with SS31 + Amlexanox + Tranilast in ARPE-19 cells. **(A)** Here, AF (4 μM, 4 h) reduces cellular ATP levels significantly in ARPE-19 cells (*p* = 0.0008; *n* = 6), which is nullified by NAC (5 mM) suggesting a role of ROS stress. **(B)** AF mediated ATP reduction in ARPE-19 (*p* = 0.02; *n* = 6) is restored by pre-treatment with SS31 + Amlexanox + Tranilast (*p* = 0.12; *n* = 6 vs. LG, control) but not with SS31 + Midiv1 + Amlexanox (*p* = 0.01; *n* = 6 vs. LG, control).

Subsequently, we examined AF-induced mitophagic flux in ARPE-19 cells using a mito-probe known as mt-Keima (Devi et al., 2013), which emits green light in mitochondria at neutral or alkaline pH (>7.0) whereas it emits red light after mitophagic flux to lysosomes at acidic pH (<5.0). Using confocal live cell imaging of ARPE-19 after mt-Keima transduction and treatment with AF, we observed mt-Keima in control cells as green filaments of mitochondria, and a lesser amount of the red mt-Keima (Figure 4A, first panel). Conversely, AF treatment increases the level of red mt-Keima in ARPE-19, indicating a mitophagy flux to acidic lysosomes (Figure 4A, second panel). Next, we tested effectiveness of several inhibitors in combination targeting different steps in the mitochon- dria-lysosome pathway (Supplementary Figures S2, S3). These include SS31 – mitochondrial antioxidant (Fivenson et al., 2017), Mdiv-1 – Drp1 fission inhibitor (Campbell et al., 2019), amlexanox – TBK1 and Optineurin/p62 inhibition (Devi et al., 2013; Manczak et al., 2019) and tranilast – NLRP3 inhibitor (Oral et al., 2017). As shown in Figure 4A (last 2 panels), we observe that the presence of Mdiv-1 reduces the level of red mt-Keima of AF but produces vesiculated mitochondria, indicating a prevention of the mitophagic flux and an accumulation of damaged mitochondria. However, a combination of SS31 + Amlexanox + Tranilast prevents AF-induced mitophagic flux as the level of green mt-Keima is maintained and the mitochondria are seen as filamentous networks. In support, the level of autophagic/mitophagic markers, LC3BII and Optineurin proteins, are increased in the present of Mdiv-1 suggesting mitophagy inhibition and reduced lysosomal degradation, which is not observed with SS31 + Amlexanox + Tranilast (Supplementary Figure S4). Furthermore, this later three drug combination normalizes mitochondrial ATP level, which is also seen with anti-oxidant NAC (N-acetylcysteine) (Figure 5), again suggesting a role for oxidative stress in mitochondrial dysregulation in ARPE-19. Therefore, this drug combination may have potential therapeutic benefits in neurodegenerative diseases.

### AF Causes Nuclear Deformation and Transcription Factor EB Translocation in ARPE-19 Cells

**FIGURE 6:**
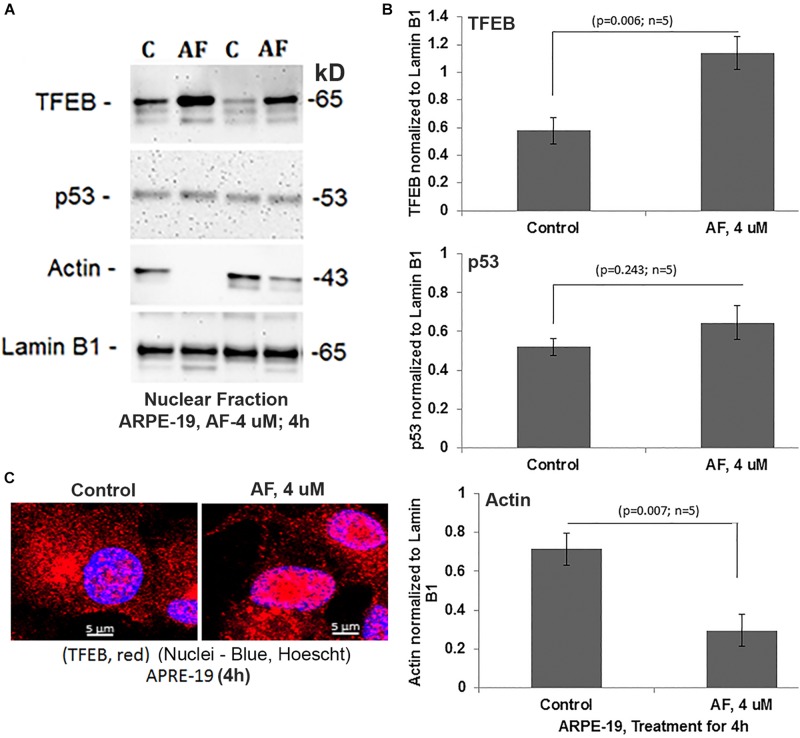
AF increases nuclear TFEB level and reduces actin in ARPE-19. **(A,B)** AF treatment (4 μM, 4 h) of ARPE-19 induces nuclear translocation of transcription factor TFEB significantly than in controls (*p* = 0.006; *n* = 5) on Western blots while that of the p53 is unchanged (*p* = 0.245; *n* = 5). Interestingly, the level of nuclear actin is significantly reduced (*p* = 0.007; *n* = 5) than in controls. All proteins were normalized to nuclear lamin B1. **(C)** Similarly, nuclear translocation of TFEB after treatment with AF (4 μM, 4 h) is also seen when examined by confocal microscopy. A representative of *n* = 3 is shown; mag. 630×.

An interesting observation with AF treatment in ARPE-19 is that the perinuclear DNA shrinks and deform when compared with the control suggesting nuclear stress (shown in Figure 4B). However, AF effects on nuclear deformation are restored by combination treatment with SS31 + Amlexanox + Tranilast, but not in the presence of Mdiv-1 (Figures 4B,C). Furthermore, AF treatment causes lysosomal destabilization shown by peripheral LAMP1-mCherry expression and membrane exocytosis in ARPE-19 cells (Supplementary Figure S5). Subsequently, one of the lysosomal membrane-associated transcription factors, TFEB, migrates from the lysosome to the nucleus as a mitophagy-lysosomal stress response (Figures 6A–C). In fact, the combination treatment with SS31 + Amlexanox + Tranilast also restores AF-induced TFEB nuclear translocation (Supplementary Figure S6), suggesting lysosomal stabilization. TFEB is involved in the synthesis of lysosomal and autophagy genes within a gene network known as the coordinated lysosomal expression and autophagy regulation (CLEAR) (Huang et al., 2018; Pan et al., 2019). On the other hand, p53 also known to response to DNA damage and cellular stress is not altered by AF treatment. However, AF treatment significantly reduces the level of nuclear actin (Figures 6A,B). Nuclear actin filaments together with lamins form the peripheral nuclear skeletal network; therefore, this observation may be related to nuclear DNA deformation or shrinkage as described above (Figure 4B) and may affect transcriptional network of TFEB.

### AF Induces Pro-inflammatory Pyroptotic Cell Death in ARPE-19 Cells

**FIGURE 7:**
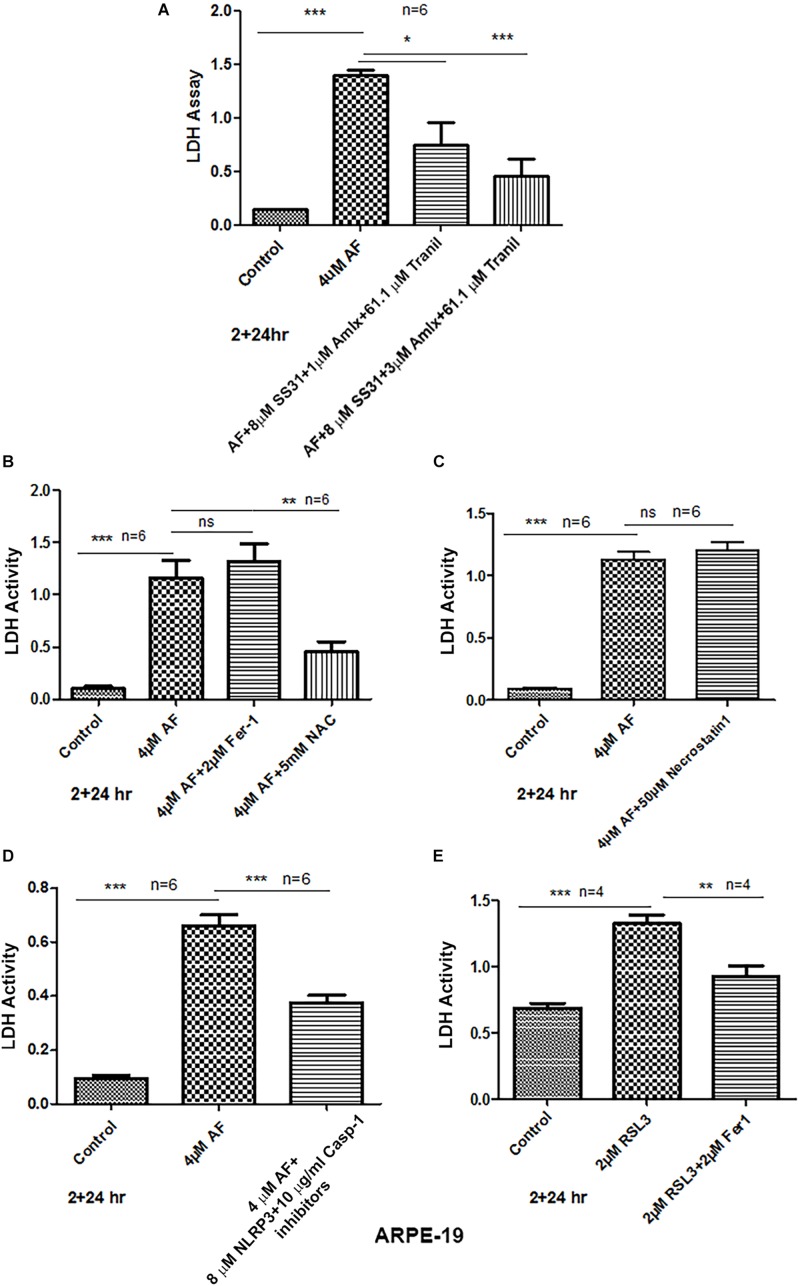
AF induces ARPE-19 death by pyroptosis. **(A)** AF (4 μM, 24 h) treatment of ARPE-19 cells induces cell death as measured by cytosolic LDH release in the culture media. AF effect is significantly countered by addition (2 h before AF) of three drugs combination SS31 + Amlexanox + Tranilast. **(B)** AF-induced LDH release is not reduced by ferrostatin-1 (inhibitor of ferroptosis, an iron-dependent cell death) but with NAC (N-acetylcysteine), suggesting the role of anti-oxidants. **(C)** Similarly, necrostatin-1 (an inhibitor of necroptosis) was without an effect on AF-mediated LDH release. **(D)** Conversely, AF-mediated LDH release is significantly reduced by a combination of NLRP3 (MCC950, 8 μM) and caspase-1 (Ac-YVAD-cmk, 10 μg/ml) inhibitors (pyroptosis inhibitors). Symbols, ^∗^*p* < 0.05; ^∗∗^*p* < 0.001; ^∗∗∗^*p* < 0.0001; *n* = 6 for all experiments. **(E)** Lastly, ferroptosis inducer RSL3 (an inhibitor GPX4) causes LDH release (ferroptotic cell death), which is significantly reduced by ferrostatin-1, further indicating that AF-mediated ARPE-19 cell death is due to inflammatory pyroptosis.

To examine further that AF causes ARPE-19 cells death, we used the release of cytosolic LDH to the culture media upon plasma membrane leakage. We did not observe ARPE-19 cell death at 4 h although mitochondrial dysregulation occurs as demonstrated above. However, as shown in Figures 6A, 7A AF increases LDH release at 24 h indicating ARPE-19 demise, which is significantly reduced by 2 h pre-incubation of a combination of SS31 + Amlexanox + Tranilast before auranofin. However, the AF-mediated LDH release is not prevented by ferrostatin 1, an inhibition of ferroptosis (Settembre and Medina, 2015), while NAC prevents cell death indicating oxidative stress mechanisms (Figure 7B). Ferroptosis is a newly identified cell death mechanism due to iron-accumulation and lipid peroxidation and inactivation of glutathione peroxidase 4 (GPX4), which detoxifies membrane lipid peroxidation (Yoshida et al., 2019). Similarly, necrostatin 1, an inhibitor of necroptosis (Forcina and Dixon, 2019), has no effect on AF-mediated ARPE-19 death (Figure 7C). On the other hand, inhibition of NLRP3 inflammasome and caspase-1 together (MCC950 and Ac-YVAD-cmk, respectively) reduces AF-mediated LDH leakage significantly in ARPE-19 (Figure 7D), indicating an inflammatory cell death mediated by caspase-1 known as pyroptosis (Hanus et al., 2016). Because mitochondria are critical for iron metabolism and its TCA cycle enzymes and ETC complexes contain iron-sulfur complexes (Pronin et al., 2019), when fluxed to lysosomes via mitophagy, there may be iron accumulation and ferroptosis. Therefore, to ensure that AF-induced ARPE-19 demise is indeed due to pyroptosis, but not by ferroptosis under the experimental conditions, we again treated ARPE-19 cells with RSL3, an inducer of ferroptosis (Lill et al., 2012). Indeed RSL3 induces LDH release in ARPE-19, which is prevented by ferrostatin 1 (Figure 7E), indicating that ferroptosis occurs in RPE cells. Furthermore, AF increases the level of gasdermin D (a pyroptosis marker) in ARPE-19 at 4 h significantly (*p* < 0.05 vs. control, 0 time), then returns to the basal level at 24 h (Supplementary Figure S7). Cytosolic gasdermin D is cleaved by activated caspase-1 and then inserts into the plasma membrane to form pores and leakage (Dubois et al., 2019). Thus, the results here suggest that AF causes cellular oxidative stress, mitochondria-lysosome dysregulation, and inflammatory pyroptotic cell death in ARPE-19.

## Discussion

**FIGURE 8:**
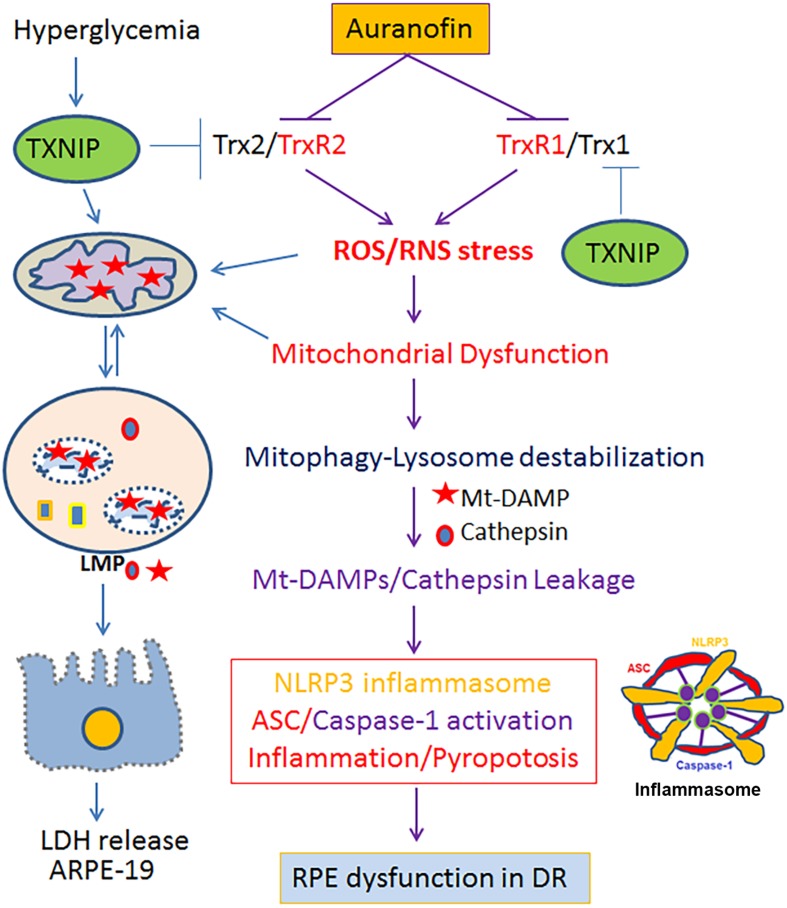
A schematic diagram depicting a potential role for the Trx/TrxR redox pathway in oxidative stress and mitochondria-lysosome axis dysfunction in RPE. Inhibition of the Trx/TrxR pathway either by auranofin on TrxR or by TXNIP on Trx under hyperglycemia (Devi et al., 2019) may lead to cellular oxidative stress, mitochondrial dysfunction, altered mitophagic flux, lysosomal damage and pro-inflammatory cell death in DR as well as in other chronic retinal neurodegenerative diseases such as AMD and RP. A combination therapy strengthening the Trx/TrxR redox system and normalizing the mitochondrial-lysosomal axis may be a potential therapeutic approach to prevent progression of various neurodegenerative diseases.

The current study identifies that the Trx redox system is critical for RPE function and an inhibition of the Trx/TrxR thiol redox by AF leads to cellular oxidative stress, mitochondrial damage, lysosomal dysfunction, and pro-inflammatory cell death as summarized in Figure 8. Furthermore, we recently published that hyperglycemia-induced TXNIP upregulation in RPE cells where Trx1 and Trx2 are inhibited causes mitochondrial damage, mitophagic flux to lysosomes and lysosomal enlargement in human RPE cells under diabetic conditions (Devi et al., 2019). Therefore, the AF effect on ARPE-19 observed here may also be similar to defects in TXNIP and Trx-thiol redox signaling defects in DR to an extent (Di Carlo et al., 2012; Devi et al., 2013; Stephenson et al., 2018). RPE oxidative stress and mitochondrial dysfunction are also seen in AMD and RP, serious blinding retinal neurodegenerative diseases (Kaarniranta et al., 2019; Zhou et al., 2019). RPE is a fully differentiated cell; therefore, they cannot dilute or distribute damaged mitochondria to daughter cells. Therefore, an efficient removal of the damage mitochondria by mitophagy and biosynthesis of new mitochondria (mitogenesis) to maintain number and bioenergetics is critically important. In addition, lysosomal function needs to be preserve for its POS phagocytosis and visual pigment recycling. Mitophagy is a protective and survival mechanism for cell viability (Pareek and Pallanck, 2018); however, too much of a mitophagic flux could lead to lysosomal overloading, enlargement, and reduced capacity to digest proteolipids causing accumulation of undigested lipofuscin and drusens in Bruch’s membrane in AMD (Dias et al., 2018; Zhou et al., 2019). Similarly, the pathogenesis of genetic neurodegenerative diseases including RP also involves RPE mitochondrial dysfunction and photoreceptor demise. As mentioned before, photoreceptors are very active neurons, which maintain a large number of mitochondria for its bioenergetics (Shu and Dunaief, 2018; Kaarniranta et al., 2019). Therefore, not only in RPE cells, mitophagy defects, and bioenergetics failure in photoreceptors may also be involved in RP etiology. Nonetheless, the role of redox imbalance and mitophagy dysfunction in RP is yet to be fully understood.

Mitochondria are the powerhouse of the cell; therefore, their dysregulation leads to excess ROS production while ATP synthesis is blunted causing bioenergetics failure in neurodegeneration (Moreno et al., 2018). In addition, damaged mitochondria release their components into the cytosol, such as mtROS and oxidized mtDNA, which are recognized as DAMPs (damage associated molecular patterns) by the cytosolic PRRs including TLRs (Toll-like receptors) and NOD-like NLRP3 inflammasomes (Zhao et al., 2019). Indeed, we demonstrate in this study that AF increases caspase-1 activity while that of the lysosomal enzyme cathepsin L is reduced, indicating an autophagic/mitophagic flux to lysosome and its destabilization (Devi et al., 2013). Cathepsins and other lysosomal acid hydrolases are important for proper digestion of the ingested cargos and recycling (exocytosis of) the digested material as nutrients (Wilkins et al., 2017). Therefore, the removal of damaged mitochondria by mitophagy is critically important to reduce cellular ROS, inflammation and premature cell demise.

Two important cellular anti-oxidant systems are the Trxs and glutathione (Samie and Xu, 2014). The Trx/TrxR works with NADPH and peroxiredoxin 3 (Prx3) to scavenge hydrogen peroxide in mitochondria and to maintain a reduced state of cysteines in a protein (Ren et al., 2017). The GSH/GPX (particularly the mtGPX4 and cytosolic GPX4) are the sole enzymes to detoxify membrane lipid peroxidation and damage (Forred et al., 2017). Therefore, these two systems work together to maintain mitochondrial health and cellular function and, therefore, a defect in one of the two systems will lead to overburden and depletion of the anti-oxidant system in mitochondria, bioenergetics failure, and mitophagy-lysosome pathway dysregulation leading to demise (Samie and Xu, 2014). Indeed, we show in this study that AF induces caspase-1 activation and cell death by pyroptosis in APRE-19 as NLRP3 and caspase-1 inhibitors prevent AF-mediated cell death. In support, we also observed an increase in gasdermin D levels by AF. Caspase-1 cleaves a 50-kD gasdermin D to a 29-kD pore forming N-terminus fragment, which is inserted into the plasma membrane causing membrane leakage and cell death by pyroptosis (Dubois et al., 2019). Recently, a role for gasdermine E in pyroptosis was implicated (Jiang et al., 2019); therefore, we will perform further studies on the role of caspase-1 and gasdermin isoforms in pyroptosis.

Furthermore, necrostatin 1 (necroptosis inhibitor) and ferrostatin 1 (ferroptosis inhibitor) are ineffective in blocking the AF-induced cell death in ARPE-19. Mitochondrial involvement in this ARPE-19 death is also evident by inhibition of the mitochondrial dysfunction and cell death by a combination of SS31 (mitochondrial antioxidant), Amlexanox (inhibits TBK1 and normalize mitophagic flux) and tranilast (which inhibits NLRP3). Therefore, these three drug combinations may be useful in the treatment of mitochondria-associated neurodegenerative diseases particularly in DR as well as in AMD and PR.

One of the important factors in the mitochondrial quality control is coordinated regulation of mitophagy (removal of damaged parts of mitochondria by autophagy as mentioned earlier) and mitogenesis (synthesis of new mitochondrial and fusion with existing mitochondria) to maintain an optimal mitochondrial number and efficient bioenergetics (Maiorino et al., 2018). One transcription factor that is critical for lysosome and autophagy gene expression is TFEB (Huang et al., 2018). TFEB is also involved in the mitochondrial biogenesis via induction of PGC1α, another important transcription factor for nuclear-encoded mitochondrial gene expression (Ni et al., 2015; Pan et al., 2019). The fact that we observe TFEB translocation to the nucleus upon AF addition to ARPE-19 indicates lysosomal destabilization. Interestingly, the nuclear actin is downregulated suggesting nuclear chromatin organization may be disturbed and, therefore, transcriptional activity may be altered (Salma et al., 2015; Lammerding and Wolf, 2016). Further studies are needed to clarify this observation. TFEB is phosphorylated at multiple sites, including Ser122, Ser142, and Ser211, by mTORC1 at the lysosomal membrane and traps TEFB in the cytosol via an interaction with 14-3-3 scaffold proteins (Hatch and Hetzer, 2016). Conversely, TFEB nuclear translocation is regulated by mucolipin 1/TRPML1, a lysosomal calcium and divalent metal ion channel (Napolitano et al., 2018). Transient release of lysosomal calcium locally by TRPML1 activates calcineurin, a protein phosphatase, which dephosphorylates TFEB sites targeted by mTORC1 and releases from 14-3-3, thereby translocating to the nucleus (Napolitano et al., 2018). Nonetheless, TRPML1 is also regulated by PI3,5 bisphosphate in the lysosomal membrane, which is synthesized by PIKFYVE kinase (Di Paola and Medina, 2019). Therefore, these kinases and phosphatases may also involve in the regulation of lysosomal function, autophagy/mitophagy flux, exocytosis, and mitochondrial biogenesis. Indeed, we observe that apilimod (Medina et al., 2015), an inhibitor of PIKFYVE, causes lysosomal enlargement and cathepsin L downregulation, while ML-SA1 (Medina et al., 2015), an activator of TRPML1, causes lysosome biogenesis and increase cathepsin L level (unpublished data). To what extent the Trx-TrxR and redox signaling regulates these kinases and phosphatases are yet to be determined in RPE and retinal neurodegenerative diseases. Nonetheless, most chronic neurodegenerative diseases involve dysregulation of mitochondrial function and lysosomal defects leading to bioenergetics failure and accumulation of undigested materials inside cells as well as extracellularly, particularly for RPE cells because they are involved in the degradation of melanosomes and phagocytosis of photoreceptor outer segments (POS) daily (Isobe et al., 2019). Thus, an accumulation of undigested oxidized lipoproteins with lysosomes will reduce lysosomal enzyme activities and enhance exocytosis of undigested material to the Bruch’s membrane (a combined basement membrane of the RPE and endothelium of the choriocapillaris), which form Drusens, yellow deposits under the retina (Isobe et al., 2019). Such an event may cause RPE dysregulation and photoreceptor dysfunction in chronic retinal neurodegenerative diseases.

## Conclusion

The present findings suggest that the Trx/TrxR redox system is critical for reducing oxidative stress, normalizing mitophagic flux, lysosome function, and prevention of pyroptotic cell death in the RPE, and therefore, in preventing retinal neurodegenerative diseases. Furthermore, the effectiveness of the therapy with three drug combinations targeting the mitochondrial ROS, mitophagic flux and NLRP3 inflammasome (SS31 + Amlexanox + Tranilast, respectively) needs to be tested in retinal neurodegenerative diseases of animal models particularly in DR. This three drugs combination may also be beneficial in the treatment and prevention of retinal neurodegenerative diseases such as AMD and RP as they also encounter mitochondrial and lysosomal dysregulation in the etiology of the diseases (Warburton et al., 2007; Bergen et al., 2019). The present study was presented as a poster at the annual meeting of the ARVO April 28, 2019 – May 02, 2019 at Vancouver, Canada.

## Data Availability Statement

All materials and data developed in this study will be made available upon request.

## Author Contributions

TY performed the cell assays, developed the methodologies, and wrote the Materials and Methods section and reviewed the manuscript. TD performed the Western blotting and immunohistochemistry. LS developed the concept, provided the guidance, and wrote the manuscript.

## Conflict of Interest

The authors declare that the research was conducted in the absence of any commercial or financial relationships that could be construed as a potential conflict of interest.
