# Supplementary material for: Auranofin Mediates Mitochondrial Dysregulation and Inflammatory Cell Death in Human Retinal Pigment Epithelial Cells: Implications of Retinal Neurodegenerative Diseases
Source: Front Neurosci. 2019 Oct 10;13:1065. doi: 10.3389/fnins.2019.01065 (PMC6795687; doi:10.3389/fnins.2019.01065)
Supplement: Supplementary file 1 [file Data_Sheet_1.pdf]

I. Supplementary Data: (Tables)

Table 1. Chemical List:

Serial No.	Name of Chemical	Name of Company	Catalog No	Lot No
1	Auranofin	Sigma Aldric	A6733	N/A
2	Amlexanox	Sigma Aldric	SML0517	N/A
3	SS31	ANASPEC	1757720	N/A
4	SS31-488	ANASPEC	1757721	N/A
5	Tranilast	Sigma Aldric	T0318	N/A
6	Mdiv-1	Sigma Aldric	M0199	N/A
7	Leu-Leu methyl ester hydrobromide (LLME)	Sigma Aldric	L7393	N/A
8	MCC950 (NLRP3 Inhibitor)	Calbiochem	5.38120.0001	3131820
9	Ac-YVAD-cmk (Caspase-1 inhibitor)	Sigma Aldric	SML0429	N/A
10	Necrostatin-1 (Necroptosis inhibitor)	EMD Millipore	CAS 4311-88-0	N/A
11.	Ferroptosis inhibitor, Ferrostatin-1	Sigma Aldric	SML0583	N/A,

Table 2A. Antibodies: Primary antibodies for Western blotting

Name	Dilution	Species	Company	Cat #
TrxR1	1:1000	mouse	MBL	K0205-3
TrxR2	1:1000	Rabbit	Cell Signalling	14714S
Trx1	1:500	Rabbit	Santa Cruz	SC-20146
Trx1	1:1000	Rabbit	Cell Signaling	2429S
Trx2	1:500	Rabbit	Santa Cruz	sc-50336
Trx2	1:1000	Rabbit	Cell Signaling	14907S
Actin	1:2000	Goat	Santa Cruz	Sc-1616
Actin	1:2000	Rabbit	Cell Signaling	4970S
TFEB	1:500	Rabbit	Cell Signaling	32361S
P53	1:500	Mouse	Cell Signaling	48818S
Lamin B1	1:1000	Rabbit	Cell Signaling	12586
LonP	1:500	Rabbit	ProteinTech	15440-1-AP

YMEIL1	1:500	Rabbit	ProteinTech	11510-1-AP
Tid1	1:1000	Mouse	Cell Signaling	4775S
PDIA6	1:1000	Rabbit	Sigma	SAB2101757
Tom20	1:1000	Rabbit	Cell Signaling	42406S
Tom20	1:1000	Rabbit	ProteinTech	11802-1-AP
Optineurin	1:500	Mouse	Santa Cruz	sc-166576
P62/SQSTM1	1:1000	Rabbit	ThermoFisher	PA5-20839
LC3B	1:3000	Rabbit	Pierce	L10382
Gasdermin D	1:1000	Rabbit	Cell Signaling	93709S

Table 2B. Secondary Antibodies: for Western blotting

**Antibody**	**Source**	**Cat#**	**Dilution**
Anti-mouse 2°ry antibody HRP	Sigma	A5278	1:2000
Anti-rabbit 2°ry antibody HRP	Sigma	A6154	1:2000
Anti-goat 2°ry antibody HRP	Santa Cruz	sc-2020	1:2000
Anti-rat 2°ry antibody HRP	Sigma	A9542	1:2000

II. Supplementary data: (Figures)

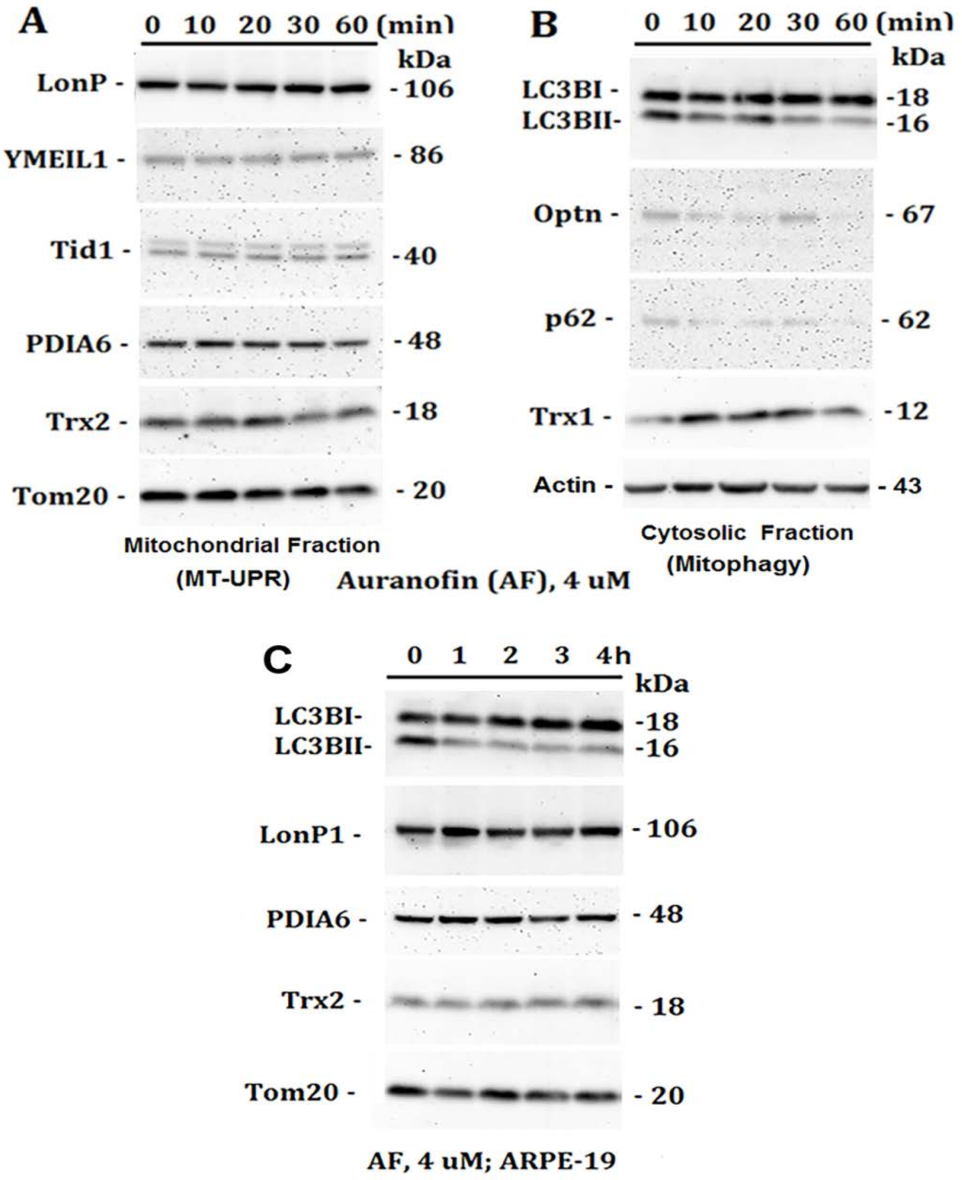

Figure S1. Auranofin induces early mitophagic response and lacks mtUPR in ARPE-19 cells. Mitochondrial oxidative stress causes protein misfolding and mitochondrial damage and mitochondrial protein stress responses including mitochondrial unfolded protein response (mtUPR) and mitophagy are activated. (A-C) However, in ARPE-19 cells, auranofin (AF, 4 μM) treatment for various time periods does not alter the level of mtUPR proteins including mitochondrial proteases (LonP1 and YMEIL1) and chaperones (Tid1, PDIA6) and antioxidant Trx2 when normalized to Tom20. (B-C) However, there is an early mitophagic response in AF treatment of ARPE-19 cells as mitophagy markers LCBII, optineurin and p62 are reduced within minutes and continues up to 4h, indicating a flux to lysosomal degradation. A representative of n=3 is shown here for all proteins.

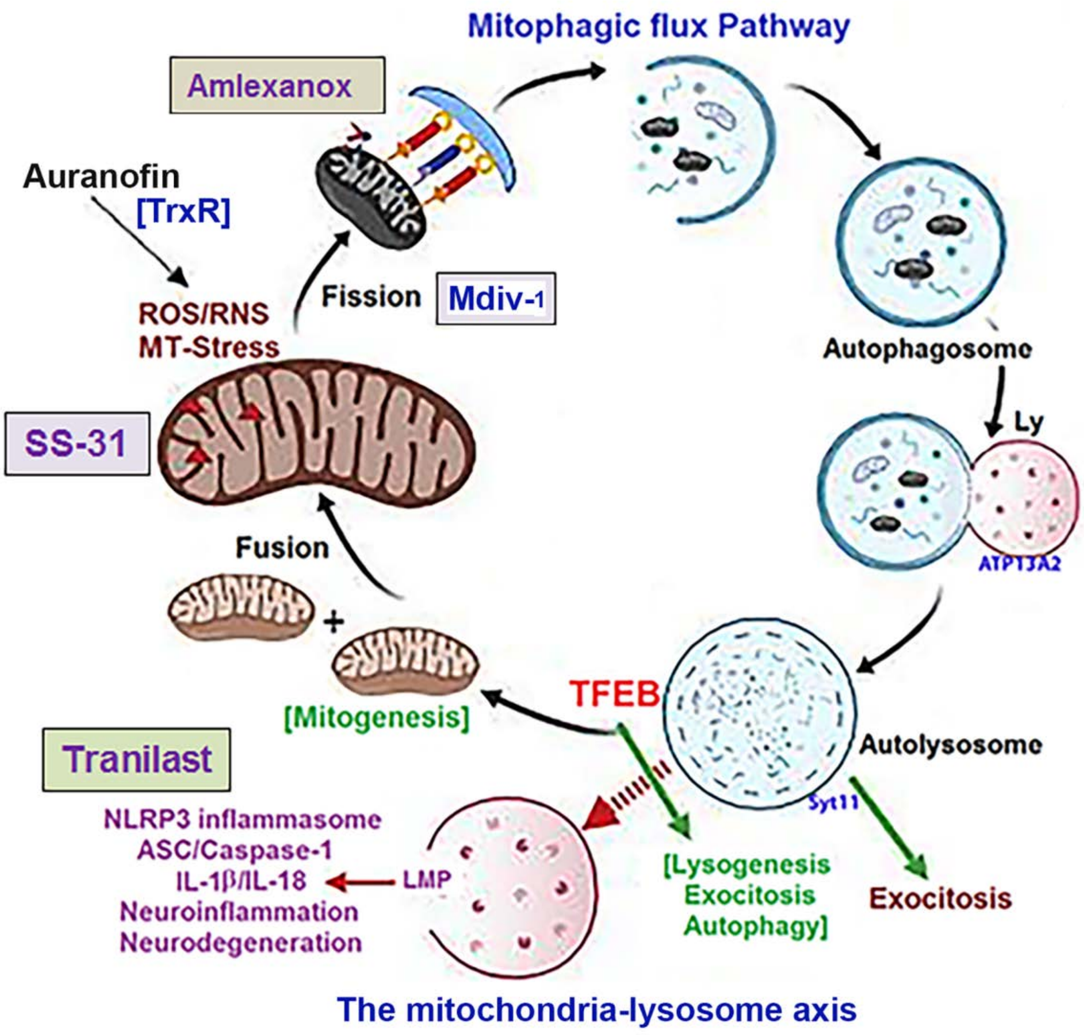

Figure S2. A potential mechanism for mitochondrial quality control by mitophagic flux and mitogenesis involving lysosome in ARPE-19 cells. Auranofin, which inhibits the redox protein TrxR, may induce mitochondrial stress, mito-fission, mitophagy flux, lysosome destabilization and inflammasome activation. Therefore, drugs that regulate the mitochondrial cycle may have protective effects in maintaining the mitophagy-lysosomal pathway under cellular stress. We tested in this study a number of drugs individually or in combinations of chemical inhibitors targeting this pathway, which includes SS31 (a mitochondria targeted anti-oxidant), Mdiv-1 (mitochondrial division inhibitor targeting Drp1), Amlexanox (an inhibitor of TBK1 kinase and optineurin/p62 phosphorylation) and Tranilast (an inhibitor of the NLRP3 inflammasome). The picture was drawn using BioRender free software.

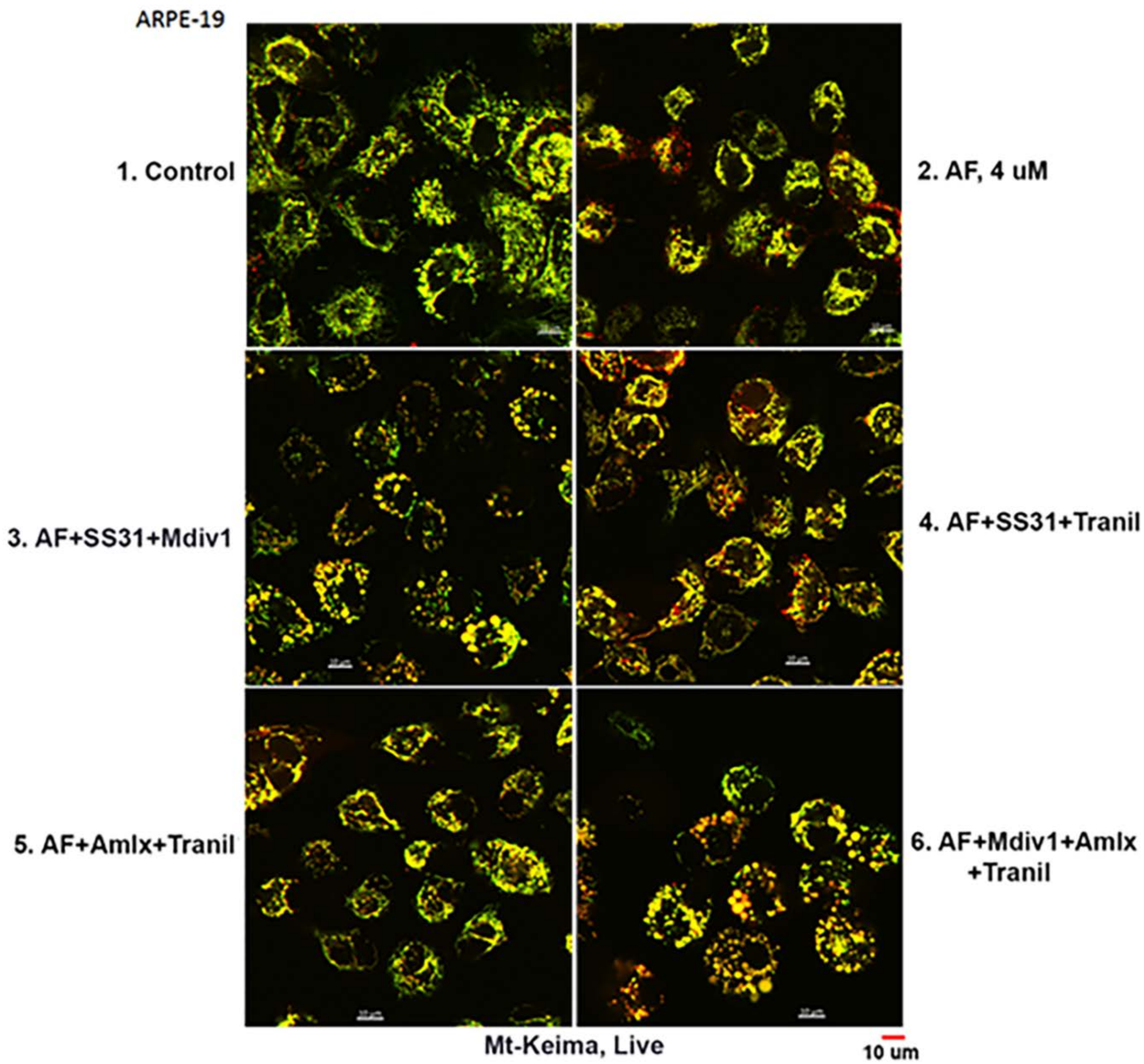

Figure S3. Auranofin induces mitochondrial dysfunction and mitophagy flux in ARPE-19. (1). In controls, mt-Keima are seen as green filaments while (2) AF (4 μM) treatment of ARPE-19 for 4h induces mitophagic flux as demonstrated by increased red mt-Keima indicating mitophagic flux. Pre-incubation with different combinations of drugs (SS31, Mdiv-1, Amlexanox or Tranilast) were test for their effectiveness against AF-induced mitophagy. However, any of them individually (not shown) or in pairs (3-5) had no significant effects on AF-induced red mt-Keima (mitophagic flux). (6) A triple combination of SS31+Mdiv-1+Amlexanox, which inhibits mito-fission (by Mdiv-1), also cause mitochondrial vesiculation and dysfunction in the presence of AF. However, a triple combination treatment with SS31+ Amlexanox+Tranilast inhibited the AF-induced mitophagy as shown in Figure 4 in main manuscript. The concentration of inhibitors used are SS31 (4 μM), Mdiv-1 (56.6 μM), Amlexanox (4 μM) and tranilast (56.5 μM). A representative of n=3 confocal images (mag 630x) is shown here.

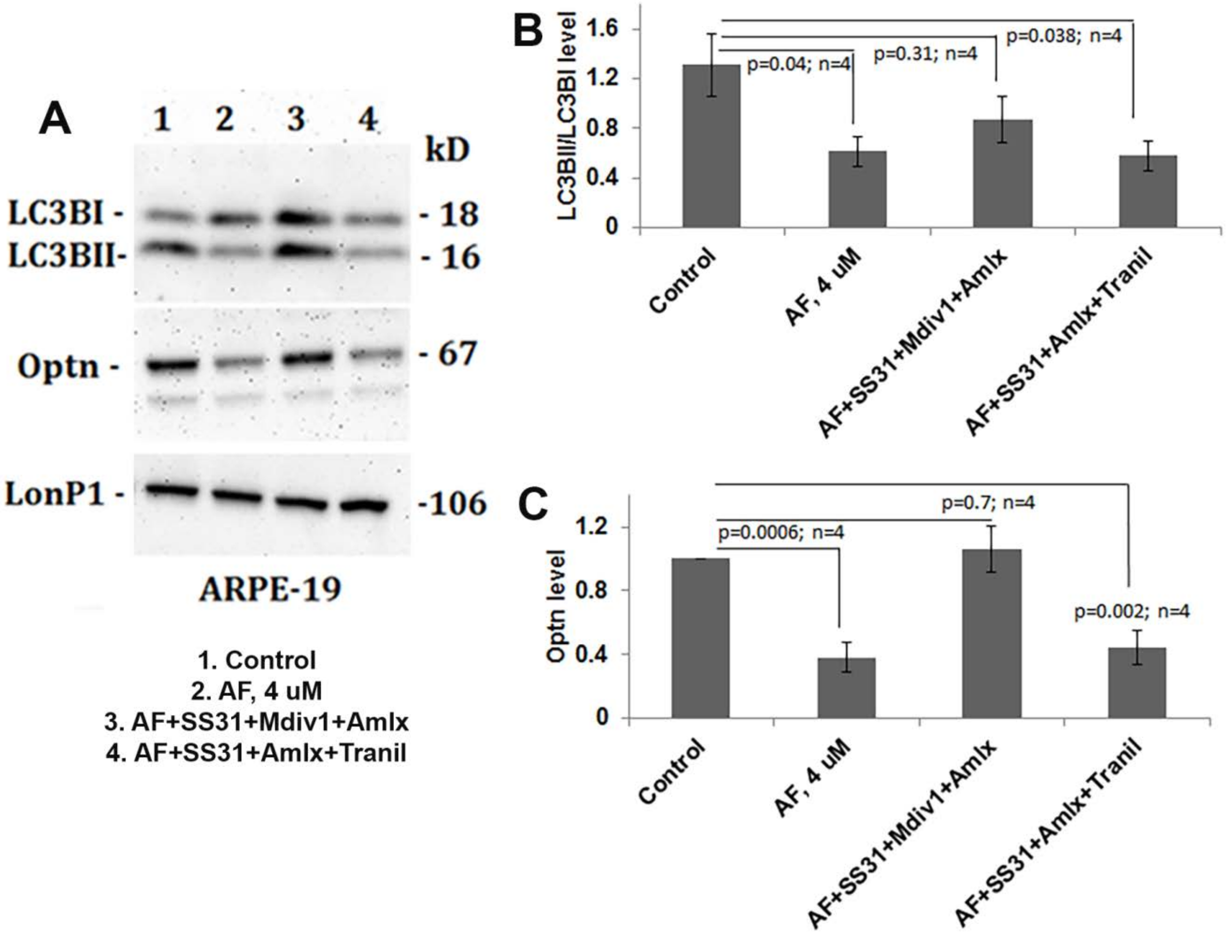

Figure 4S. Auranofin induces mitophagy flux in ARPE-19. Auranofin significantly reduces the level of mitophagy markers such as double membrane LC3BII (A-B) and adaptor Optineurin (A-C), which was normalized to mitochondrial protein LonP1. Pre-incubation with SS31+Mdiv-1+Amlx increases the level of LC3BII/I and Optineurin in the presence of AF suggesting mitophagy inhibition. This is due to the presence of and inhibition of mitophagic flux by Mdiv-1. On the other hand, SS31 + Amlexanox + tranilast still maintain an optimal mitophagic flux (reduced LC3BII/I and Optineurin as seen in lane 4, A). This observation suggests maintenance of the mitochondrial homeostasis even in the presence of AF. The concentration of inhibitors used are SS31 (4 μM), Mdiv-1 (56.6 μM), Amlexanox (4 μM) and tranilast (56.5 μM).

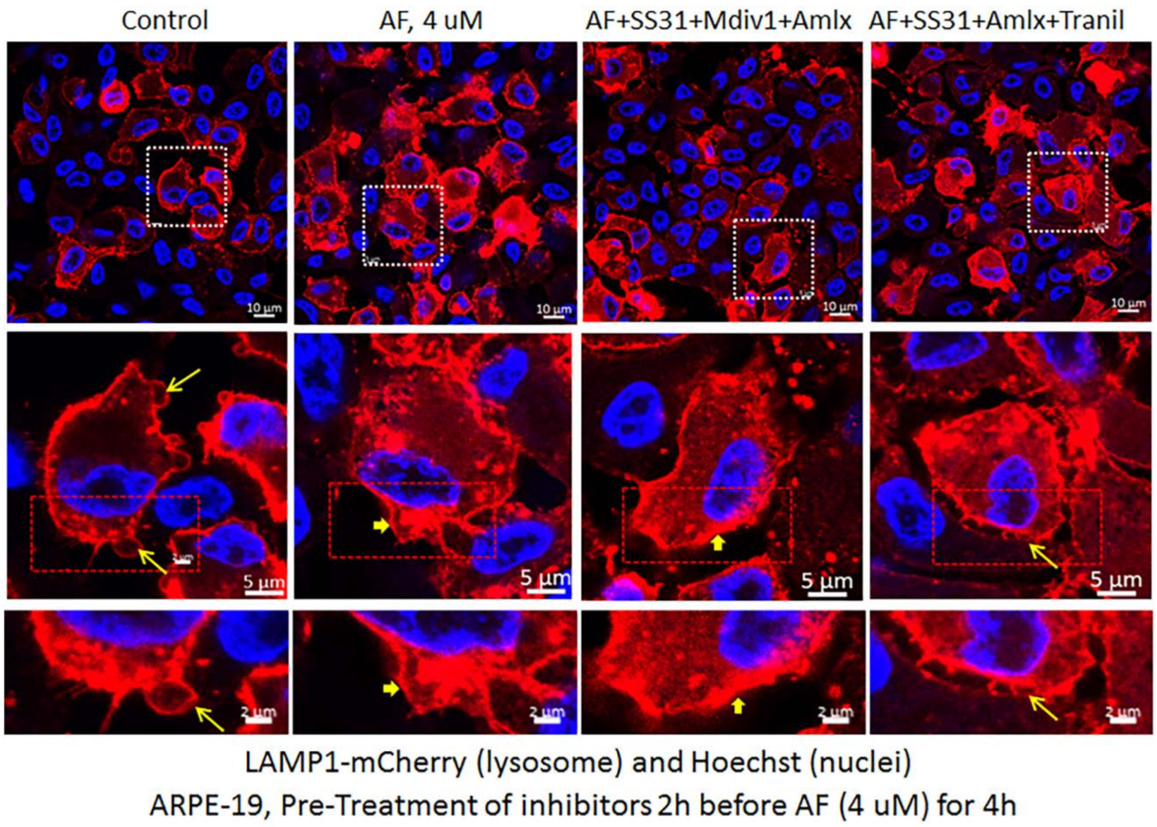

Figure S5. AF causes lysosomal destabilization in ARPE-19. We used lysosomal membrane protein LAMP1-mCherry to monitor lysosomal morphology after auranofin treatment in ARPE-19 (ref. 28). Ad-CMV-LAMP1-mCherry was transduced for 3 days to expression LAMP1-mCherry and treated with AF (4 μM, 4h). In controls, we observed lysosomal exocytosis at the plasma membrane (longer/narrow arrows), which is absent after treatment with AF (shorter/larger arrows). Preincubation with SS31+Mdiv-1+Amlexanox has no effect on LAMP1-mCherry (shorter/larger arrows at 3rd panel) while inclusion of SS31+Amlexanox+Tranilast restored LAMP1-mCherry exocytosis similar to that observed in controls (longer/thinner arrow). The concentration of inhibitors used are SS31 (4 μM), Mdiv-1 (56.6 μM), Amlexanox (4 μM) and tranilast (56.5 μM).Details of lysosomal fusion, fission and exocytosis in ARPE-19 cells in lysosomal stress will be studied in a separate manuscript. Lysosome dysfunction leads to many lysosomal storage disorders (LSD), which also affect the neurons and the central nervous system (Kielian T. Lysosomal storage disorders: pathology within the lysosome and beyond. Neurochem. 2019 Mar;148(5):568-572. doi: 10.1111/jnc.14672). A representative of n=3 confocal images is shown; mag. 630x.

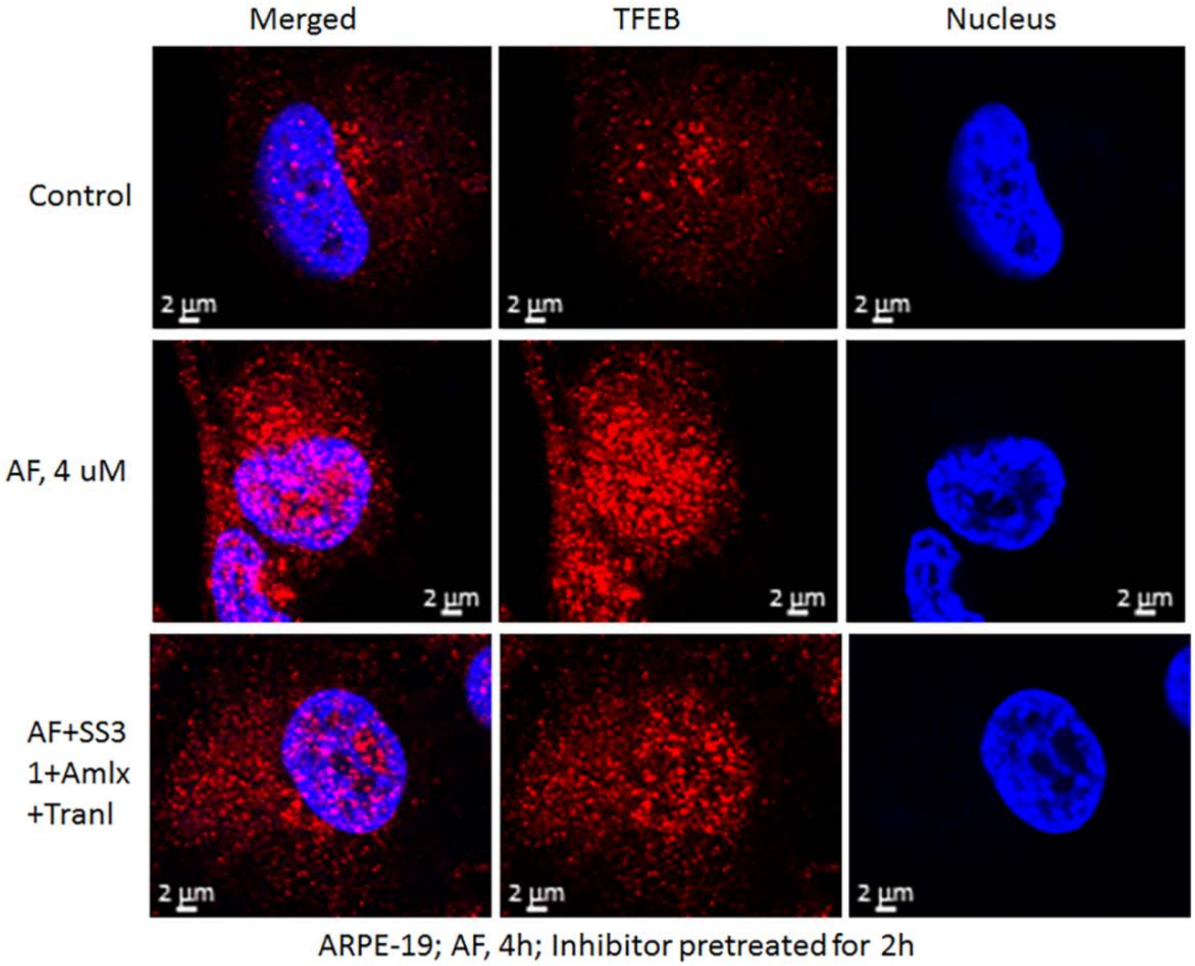

Figure S6. AF increase nuclear TFEB transloation and inhibited by triple combination drug treatment in ARPE-19. AF treatment (4 µM, 4h) of ARPE-19 induces nuclear translocation of transcription factor TFEB when compared with the control when examined under confocal microscopy. On the other hand, preincubation with a combination of SS31 (4 µM) +Amlexanox (4 µM)+Tranilast (56.5 µM) reduces the AF-induced TFEB nuclear localization when compared to AF alone, suggesting a reduction in lysosomal stress in ARPE-19. A representative of n=3 is shown; mag. 630x.

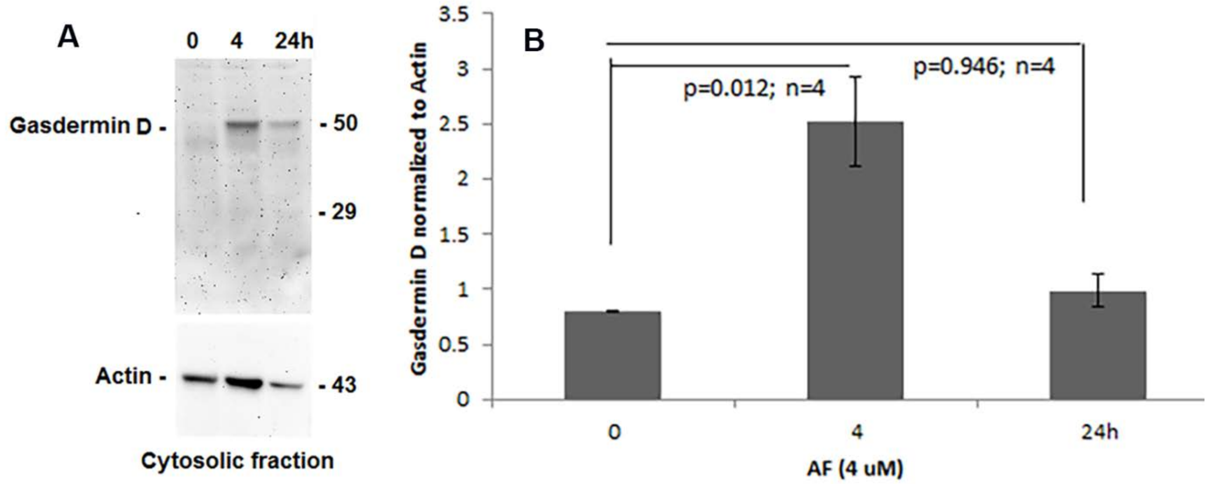

Figure S7. AF increases Gasdermin D level in ARPE-19. ARPE-19 cells were treated with AF (4 μM) for 4 or 24 h. Cells were harvested and cytosolic fractions were prepared. On Western blots, Gasdermin D and actin were probed using respectively primary antibodies. We observed that AF increases Gasdermin D significantly at 4h and then reduces at 24h. Gasdermin D is cytoplasmic protein of 50 kD, which is cleaved by caspase-1 to generate a 29-kD N-terminus fragment that is then inserted into the plasma membrane to form a pore and membrane leakage. Therefore, gasdermin D is considered as a marker for caspase-1 dependent cell death by pyroptosis. The fact that there is an increase in cytosolic gasdermin D at 4h and a decrease at 24h may be due to integration in the plasma membrane in pyroptosis. However, we did not perform a plasma membrane fractionation and determine the 29-kD fragment. Alternatively, the decrease in cytosolic gasdermin D at 24h could be due to protein degradation at late stages of pyroptosis. These alternative mechanisms will need further studies in future.
